# 32 Element Piezoelectric Micromachined Ultrasound Transducer (PMUT) Phased Array for Neuromodulation

**DOI:** 10.1109/ojuffc.2022.3196823

**Published:** 2022-08-05

**Authors:** PANNAWIT TIPSAWAT, SHEIKH JAWAD ILHAM, JUNG IN YANG, ZEINAB KASHANI, MEHDI KIANI, SUSAN TROLIER-MCKINSTRY

**Affiliations:** 1 Department of Materials Science and Engineering and Materials Research Institute, The Pennsylvania State University, University Park, PA 16802 USA; 2 Department of Electrical Engineering, The Pennsylvania State University, University Park, PA 16802 USA

**Keywords:** Neuromodulation, focused ultrasound stimulation, piezoelectric micromachined ultrasound transducer, thin film, phased array

## Abstract

Interest in utilizing ultrasound (US) transducers for non-invasive neuromodulation treatment, including for low intensity transcranial focused ultrasound stimulation (tFUS), has grown rapidly. The most widely demonstrated US transducers for tFUS are either bulk piezoelectric transducers or capacitive micromachine transducers (CMUT) which require high voltage excitation to operate. In order to advance the development of the US transducers towards small, portable devices for safe tFUS at large scale, a low voltage array of US transducers with beam focusing and steering capability is of interest. This work presents the design methodology, fabrication, and characterization of 32-element phased array piezoelectric micromachined ultrasound transducers (PMUT) using 1.5 *μ*m thick Pb(Zr_0.52_ Ti_0.48_)O_3_ films doped with 2 mol% Nb. The electrode/piezoelectric/electrode stack was deposited on a silicon on insulator (SOI) wafer with a 2 *μ*m silicon device layer that serves as the passive elastic layer for bending-mode vibration. The fabricated 32-element PMUT has a central frequency at 1.4 MHz. Ultrasound beam focusing and steering (through beamforming) was demonstrated where the array was driven with 14.6 V square unipolar pulses. The PMUT generated a maximum peak-to-peak focused acoustic pressure output of 0.44 MPa at a focal distance of 20 mm with a 9.2 mm and 1 mm axial and lateral resolution, respectively. The maximum pressure is equivalent to a spatial-peak pulse-average intensity of 1.29 W/cm^2^, which is suitable for tFUS application.

## I. INTRODUCTION

LOW intensity transcranial focused ultrasound stimulation (tFUS) is rapidly growing as a noninvasive neuromodulation treatment with a millimeter spatial resolution compared to modalities such as transcranial magnetic, direct current, or alternating current stimulation [1]–[3]. Multifarious therapeutic applications of tFUS in animal models such as mice, rats, sheep, and nonhuman primates have been reported over the past decade, most of which target fundamental neuroscience studies [4]–[14]. This was followed by a recent surge in application of tFUS neuromodulation in human subjects, largely for clinical neuroscience studies [15]–[24]. Moreover, tFUS has already been associated with other extant imaging technologies, such as magnetic resonance imaging (MRI) or brightness mode (B-mode) ultrasound imaging which promise further improvement in the accuracy of neuromodulation in both basic and clinical neuroscience applications [1], [7].

Conventionally, tFUS experiments utilize single element ultrasound (US) transducers with a relatively large diameter (tens of millimeters) and a fixed focal spot. A precise manipulator is required to mechanically alter the focal spot to stimulate different brain regions, which is a major shortcoming of conventional tFUS systems. However, this limitation can be overcome by leveraging the electronic beam focusing and steering capabilities of optimally designed US phased arrays. In particular, a US phased array enables electronically controllable stimulation over a large tissue volume, i.e., large-scale stimulation.

In recent years, several US phased array transducers designed for tFUS have been reported, especially diced ceramic transducers and capacitive micromachined ultrasound transducers (CMUTs). For example, a ring-shaped 32-element US phased array (CMUT), operating at 80 V_AC_ with a 100 V_DC_ offset at 183 kHz, yielded a maximum 52 kPa pressure generating a temporal-average spatial-peak acoustic intensity (*I_SPTA_*) of ~55.4 mW/cm^2^. The CMUT successfully stimulated motor cortical areas in freely moving mice [25]. A wearable 2D array device (CMUT) integrated with a complementary metal-oxide-semiconductor (CMOS) chip has been developed in [26] that generated an acoustic pressure output of ~ 575 kPa while operating at 2 MHz with 60 V_AC_. A 26 × 26.2D phased array using 267 *μ*m thick PZT-5A piezoelectric ceramics as transducers has been fabricated on a CMOS chip, delivering an acoustic pressure of 40 kPa at 5 V excitation at 8.4 MHz [27]. Moreover, a relatively thinner bulk PZT transducer was presented in [28] where the 16-element array with a 40 *μ*m thick PZT layer on a silicon on insulator (SOI) wafer could achieve a peak intensity up to 1.1 W/cm^2^ with an input of 66 V.

Despite these recent efforts, piezoelectric micromachined ultrasound transducers (PMUT) with piezoelectric thin films offer advantages in device miniaturization, high bandwidth and sensitivity, and compatibility with the front-end electronic integration [29]. But the use of thin film PMUTs for tFUS applications has not yet been reported.

A design methodology for geometry optimization for large-scale US neuromodulation has been proposed in [30]. The design method maximizes a figure of merit (FoM) that simultaneously considers the total input power consumed by the array, the peak pressure engendered at the focal spot, and the overall focal volume defined by the half power beam width. In brief, the $\mathrm{FoM}=P/(\surd \mathrm{NaL}\times \sqrt[{3}]{V})$, where *P* is the spatial peak US pressure output (for the same voltage amplitude across each element), *V* is the half-power-beam-width focal volume at the focal spot, *N* is the number of array elements, *a* is the element width, and *L* is the element length. It is worth mentioning that *NaL* has been included in the FoM to account for a constant total input power to the array as *N*, *a*, and *L* are swept during the optimization. The described design methodology balances among the input power, the generated pressure, and the spatial resolution, thereby leading to an optimally performing phased array design. In that work, a 16-element linear phased array transducer at 833.3 kHz was fabricated using bulk PZT, outputting 1.15 MPa peak pressure within a beam having a lateral resolution of 1.6 mm at 12 mm focal distance with a 150 V_PP_ excitation voltage.

In this article, a comparable design methodology for phased array transducers has been applied to PMUT with a 1.5 *μ*m thick PZT thin film, specifically intended to be driven by relatively lower voltages (<20 V), while generating an acoustic pressure comparable to that of bulk PZT arrays with a comparable input power consumption. An US PMUT array operating in the bending mode was designed, fabricated, and characterized for the neuromodulation application. The design optimization of the array will be discussed in Section II. The detailed fabrication method will be described in Section III. The measurement results will be discussed in Section IV, with conclusions in Section V.

## II. PMUT ARRAY DESIGN

**FIGURE 1.**
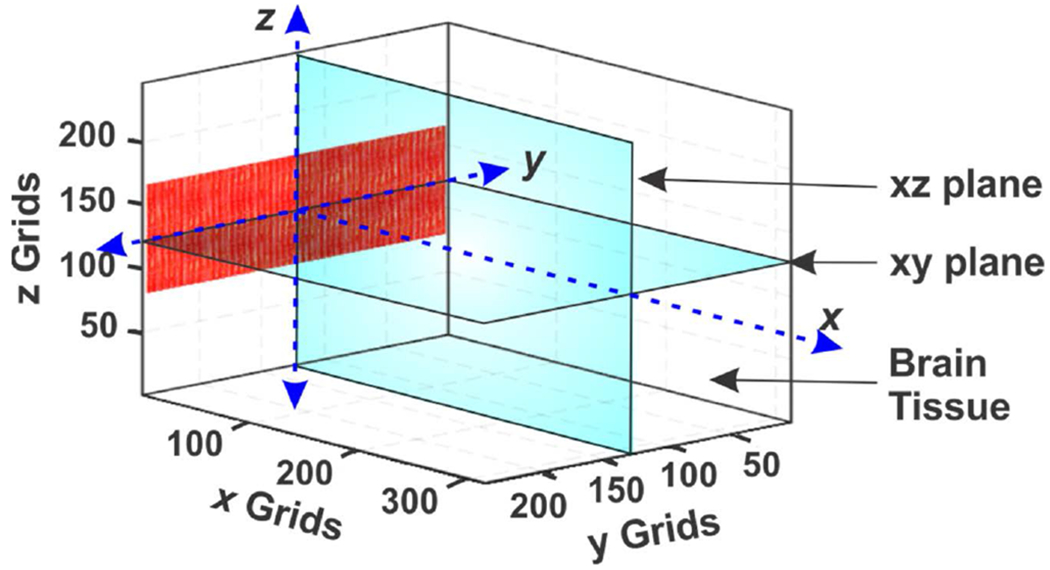
A linear transducer positioned in a 3D grid space defined in k-Wave. The xy and xz planes are defined as the sensors. The medium has properties like brain tissue.

Based on the design procedure presented in [30], the geometries, i.e., element length (*L*), element pitch (*d*), element width (*a*), and the number of elements (*N*) of an US phased array were optimized using k-Wave, which is an open-source MATLAB toolbox. Since the element thickness is not considered in k-Wave, the optimization process for a bulk array in [30] can still be used for the 2D geometry optimization of the PMUT array. The array was designed for tFUS of a rat’s brain with high FoM while having the least off target stimulation effects. For this design, the following assumptions were made: 1) Array aperture *D* and element length *L* were limited by the nominal dimensions of a rat’s head (*D*_*max*_ = *L*_*max*_ = 25 mm); 2) Focal distance, *F*, was set to 20 mm so that it can cover the standard depth of a rat’s brain [31]; 3) the resonant frequency, *f,* was set to ~ 1 MHz considering the high attenuation encountered by higher frequency US penetrating the scalp, skull, and brain tissue [32]; 4) the maximum steering angle (*θ_s,max_*) was ±60°; 5) the minimum kerf of 96.5 *μ*m (λ/16, where λ is the wavelength) was set as the grid resolution of the k-Wave simulations; 6) *H*(*y*) < 0.7, where *H*(*y*) is an equivalent to the directivity function and is defined as the ratio of the peak output pressure that occurred on a line parallel to the ***x*** axis (which corresponds to different ***y*** values with ***z*** = 0 in Fig. 1) to the peak output pressure at the focal spot (which corresponds to a single point in the whole ***xy*** plane) [30]. Since the US beam area is defined by half of the maximum power, which corresponds to ~0.7 of peak pressure, a threshold of 0.7 is reasonable for *H*(*y*).

Fig. 1 shows the 3D k-Wave simulation setup with a 314 × 314 × 246 grid space having the properties of brain tissue. The spatial and temporal grid resolutions were set to 96.5 *μ*m (λ/16) and 18.75 ns (determined by the time needed to travel the diagonal of the grid space). To abolish artefacts due to reflections from the grid boundaries, a perfect matching layer (PML) of 0.48 mm thickness was added to the boundaries. The sound speed, mass density, and the attenuation coefficients were set based on [33]. Overall, each simulation took ~92 minutes on a regular desktop.

**FIGURE 2.**
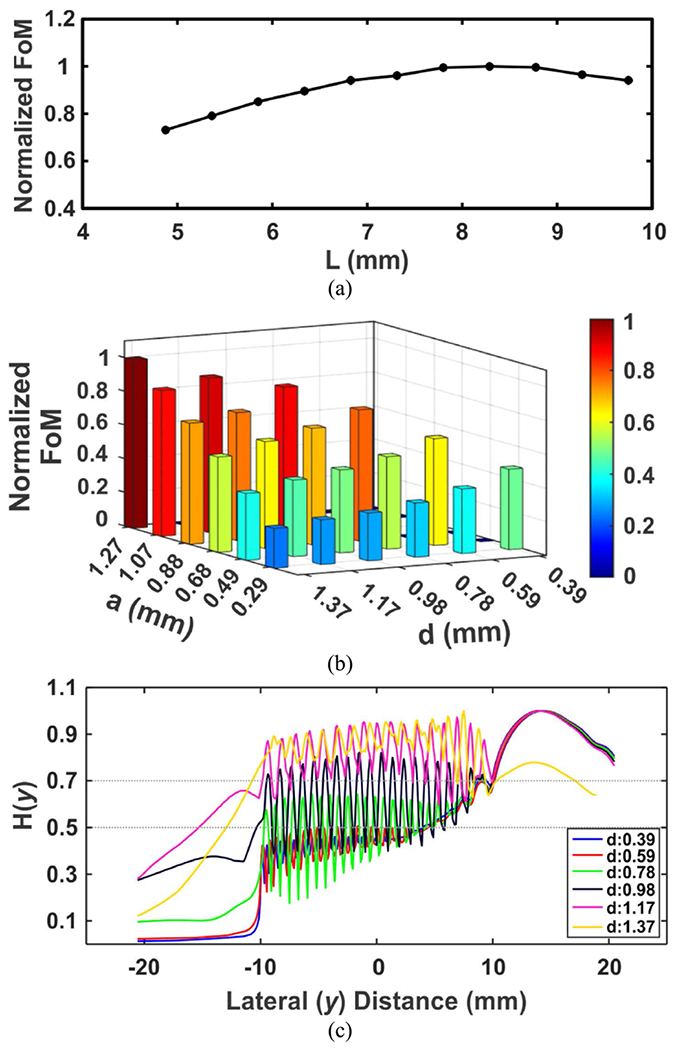
Optimization results for the phased array targeting *F* = 20 mm and *H*(*y*) < 0.7 at *θ*_*s*_ = 60°. (a) First iteration showing the normalized FoM vs. *L*. (b) First iteration showing the normalized FoM vs. *d* and *a*. (c) *H*(*y*) at different *d* (with the optimum *a*).

Fig. 2 demonstrates the results acquired by implementing the optimization method described in [30]. Setting the initial values of the interelement spacing, *d* = λ/2 = 0.78 mm, and the element width, *a* = *d*/2 = 0.385 mm, first the element length, *L* was optimized for the maximum FoM. The optimum value of *L* was found to be 8.3 mm as depicted in Fig. 2a. Next, *d* and *a* were simultaneously swept to maximize the FoM with the optimum *L*. Fig. 2b depicts a 3D plot of normalized FoM as a function of *d* and *a*. The highest FoM was found at *d* = 1.37 mm with *a* = 1.27 mm. However, there is a trade-off between having a larger *d* and steering the beam with smaller grating/side lobes (i.e., the focused beam at *F* = 20 mm could yield *θ_s,max_* = 60°). Although from Fig. 2b it is apparent that larger *d* (having a larger *a*) tends to maximize the FoM, Fig. 2c demonstrates that *d* should be limited by a threshold based on *H*(*y*). One should take note that *H*(*y*) is equivalent to the directivity function (which should be considered to either avoid off-target stimulation or to keep it under a particular threshold). It is worth noting that *L*, *a*, and *d* are the independent sweeping parameters, whereas *N* and *D* are dependent on *d* with the relation of *D* = *N* × *d* – *kerf*. The optimization of *N* and *D* as a dependent parameter has been discussed in detail in [30]. By applying the iterative optimization procedure, the optimum array geometries were found to be *L* = 8.3 mm, *d* = 0.78 mm, *a* = 0.68 mm, and *N* = 32.

**FIGURE 3.**
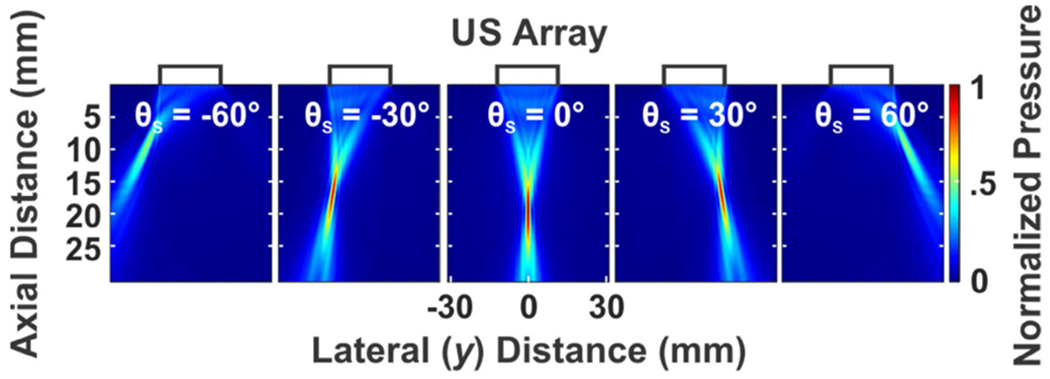
Simulated beam steering capability of the optimum array in Table 1.

Table 1 summarizes the optimum design parameters achieved by the iterative method as well as the geometry of the fabricated array side by side. With the optimum array geometry, the beam steering capability (at *F* = 20 mm with *θ*_*s*_ of −60° to 60°) of the array has been checked in simulations as depicted in Fig. 3.

An analytical method was used to design the active PMUT resonator area defined by the silicon etch trench through the Si handle wafer. The fundamental frequency of a clamped rectangular plate is given by: [34]

(1) $$2\pi f_{r}=\left(\frac{1}{L^{2}}+\frac{1}{a^{2}}\right)\cdot \sqrt{\frac{D_{r}(E,v)}{\rho_{i}t_{i}}}$$

where *L* and *a* are the length and width of the rectangular plate, *D_r_* is flexural rigidity, *ρ_i_* is the density of the *i*^*th*^ layer of a thin film stack, and *t_i_* is the thickness of the *i*^*th*^ layer. The thin film stack consisted of the buried oxide layer, Si layer, thermally deposited SiO_2_, and the piezoelectric stack: TiO_2_-Pt as a bottom electrode, PZT, and Ti-Pt as a top electrode. To achieve 1 MHz resonant frequency in water, a rectangular plate of 8000 *μ*m in length and 130 *μ*m in width with 2 *μ*m silicon layer was selected. Then, the individual PMUT element was designed by combining two approaches. The PZT bar dimension was based on the 8.3 × 0.78 mm dimension found from the design methodology above, while the active piezoelectric area was defined by the trench dimension calculated analytically to be ~8000 × 130 *μ*m. Since the trench area is smaller than the PZT bar, three parallel trenches were fitted into the single element to maximize the output power.

## III. PMUT FABRICATION AND CHARACTERIZATION

### A. FABRICATION

**FIGURE 4.**
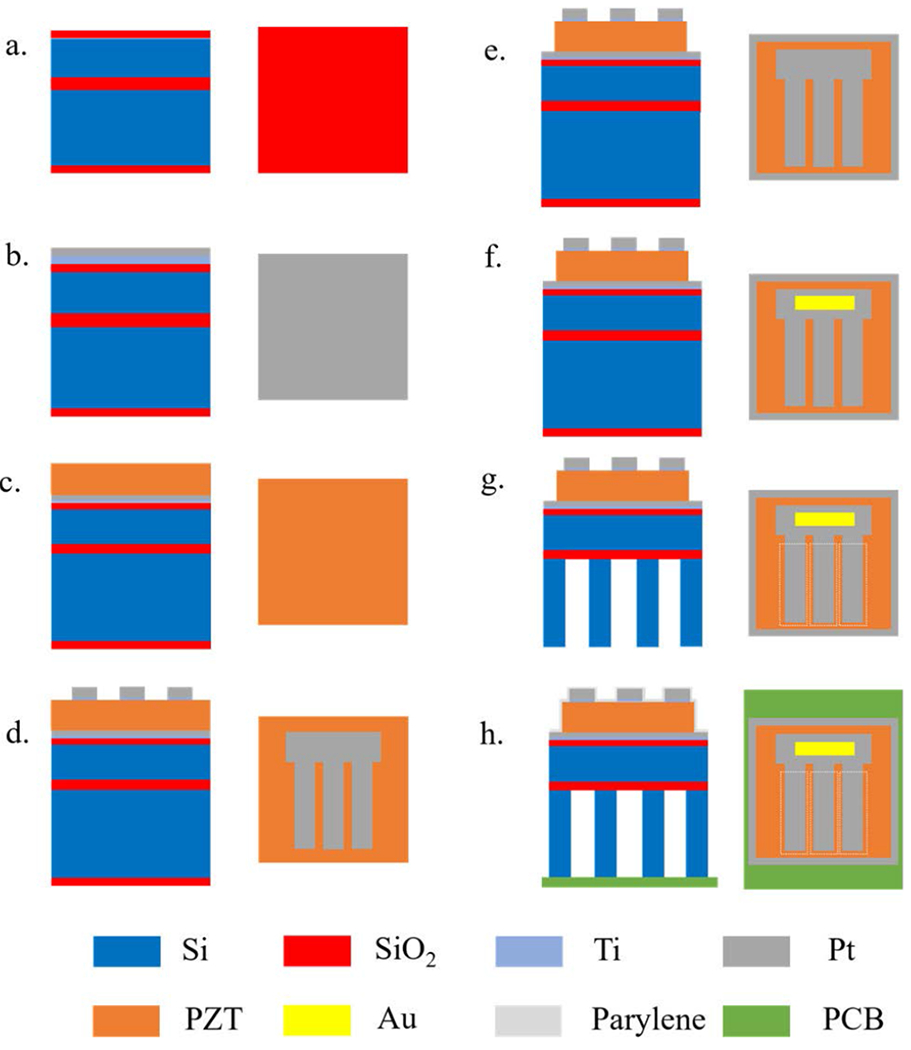
Schematic process flow for PMUT fabrication: a) SOI substrate with 2 *μ*m of silicon on the device side, b) bottom electrode deposition, c) PZT spin coating, d) top electrode deposition and patterning, e) PZT patterning, f) contact pad deposition and patterning, g) back side silicon trench etching, and h) PCB mounting, wire bonding, and waterproofing with a polymer coating.

The microfabrication process flow used to fabricate the PMUT is illustrated in Fig. 4; the process is similar to that previously reported [35]. Each individual element was patterned into 8570 × 682 *μ*m PZT resonator bar with three parallel released trenches of 130 *μ*m width with 60% top electrode coverage [36]–[38]. The element pitch is *λ*/2 (780 *μ*m). The fabrication was based on a silicon on insulator (SOI) wafer with a 2 *μ*m Si device thickness, 3 *μ*m buried oxide layer and 400 *μ*m handle thickness (Ultrasil Corp., CA, USA). The SOI wafer was coated with 560 nm SiO_2_ by wet thermal oxidation on both sides.

Highly {111} oriented Pt bottom electrodes were prepared as described by [39]. In short, 30 nm of Ti was DC sputtered at room temperature, followed by rapid thermal annealing (RTA) with 10 slpm O_2_ atmosphere at 700 °C for 15 minutes to form TiO_2_. The Pt was DC sputter deposited at a substrate temperature set at 600°C.

**FIGURE 5.**
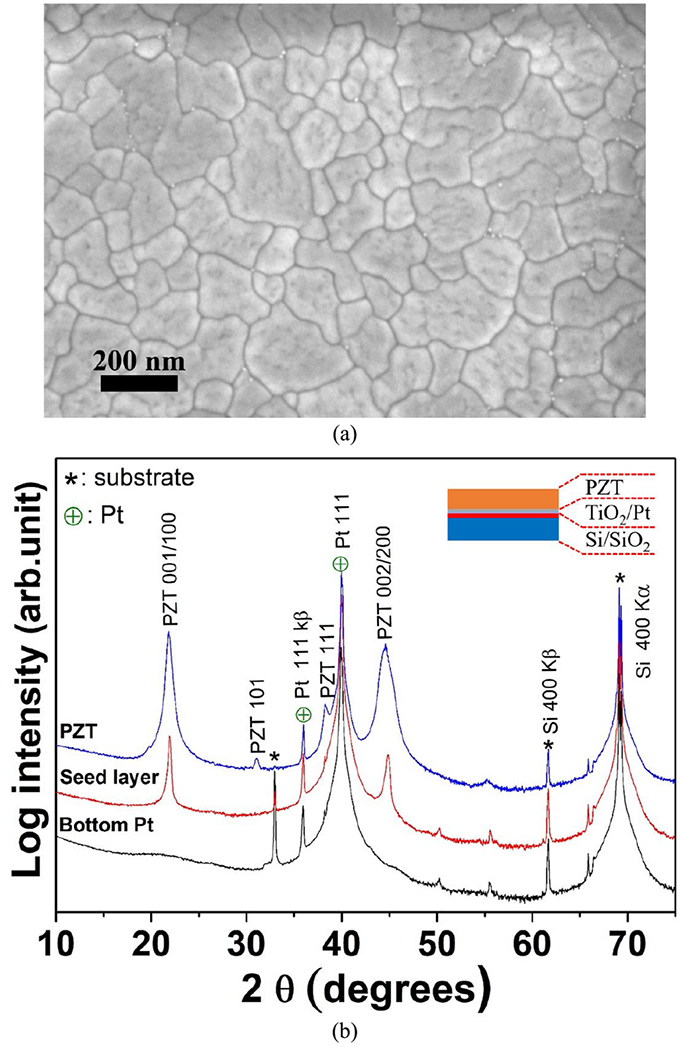
(a) The FESEM image and (b) the XRD patterns of a 1.5 *μ*m thick {001} PZT thin film showing that well oriented PZT was achieved. The very fine white particles observed predominantly near triple points in (a) are the pyrochlore/fluorite second phase.

To maximize the piezoelectric response, it is important to obtain a highly {001} oriented PZT thin film. Hence, 2% Nb doped PbZr_0.44_Ti_0.56_O_3_ sol gel solution (Mitsubishi Materials Corp., Hyogo, Japan) was employed as a seed layer. The seed layer solution was spun at 3500 rpm, pyrolyzed at 200°C for 2.5 minutes and crystallized in RTA at 700°C for 1 min in 2 slpm O_2_ atmosphere, following the work in [40], [41]. Then, a 2% Nb doped PbTi_0.52_Zr_0.48_O_3_ was deposited using 0.4 Molar 2-MOE based solution by spin coating at 1500 rpm for 45 seconds, followed by pyrolysis at 225 °C and 400 °C for 2 and 3 minutes, respectively [42]. The crystallization was done in a rapid thermal annealer at 700 °C for 1 min with 2 slpm of O_2_. The process was repeated until the desired thickness of 1.5 *μ*m was achieved. After that, the PZT thin film was characterized with a field-emission electron microscope (FESEM; Carl Zeiss Microscopy LLC., White Plains, NY, USA) and x-ray diffraction (XRD; Malvern Panalytical Ltd., Malvern, UK) as shown in Fig. 5. With the {001} and {002} peaks shown at 21.9° and 44.6° of 2*θ*, these results confirmed that the PZT thin film was predominantly {001} oriented perovskite with a slight amount of surface pyrochlore along the grain boundaries. Finally, a 2 nm Ti adhesion layer and 100 nm of Pt were DC sputtered without breaking vacuum as the top electrode.

The Pt top electrode and PZT blanket films were then patterned into individual elements using an inductively coupled plasma – reactive ion etching (ICP-RIE) Ulvac NE-500 system (Ulvac, Inc., Kanagawa, Japan). The top electrode was patterned using a 2 *μ*m thick SPR 955 photoresist. The etching process was adapted from [43] and conducted in an ICP-RIE system in 30 sccm of Cl_2_ and 40 sccm of Ar at 700 W ICP and 100 W RIE power. The photoresist was then removed by immersing the sample into Baker PRS3000 photoresist remover at 80 °C for 30 minutes, followed by 3 minutes in an oxygen plasma. For PZT etching, 13 *μ*m thick AZ4620 was spun at 4500 rpm for 45 seconds and soft baked at 90 °C and 105 °C for 1 and 3 minutes, respectively. Before exposing the photoresist, the spun photoresist was rehydrated in air for an hour and exposed at 100 mJ/cm^2^ for 8 cycles with 30 second delay times between each step. Then, the exposed photoresist was developed in 1:4 AZ400K developer for 4 minutes. The ICP-RIE was performed in ICP-RIE system with 3.5 sccm of Cl_2_, 7 sccm of CF_4_, and 10 sccm of Ar with 600 W ICP and 150 W RIE power. The finished sample was cleaned using a similar process in a photoresist remover, and an oxygen plasma as mentioned earlier.

**FIGURE 6.**
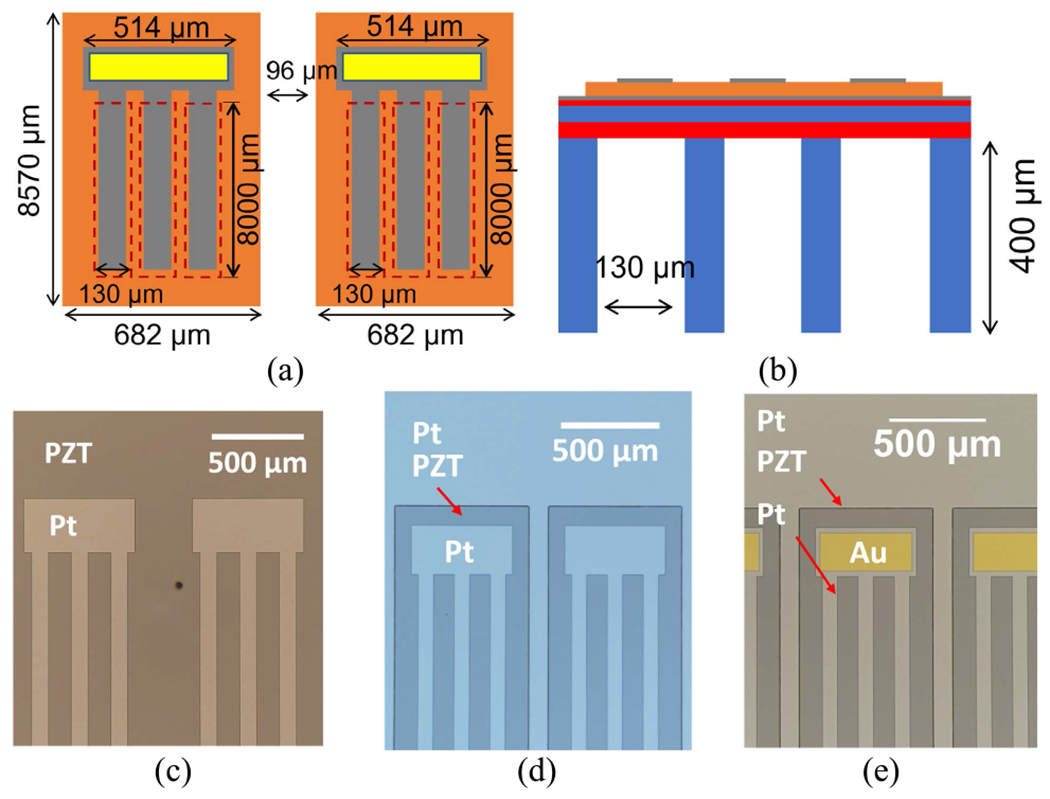
Schematics of the device with the dimensions shown in (a) cross section and (b) top view. The element width is 514 μm. Optical microscope images of device side pattern showed (c) top electrode pattern and (d) PZT pattern after ICP-RIE, and (e) Au pad after wet etching.

Fig. 6c and 6d shows microscope images of individual elements after top electrode and PZT patterning, respectively. 500 nm of Au was deposited by DC sputtering and patterned by wet etching using Au etchant TFA type (Transene Company, Inc., MA, USA) at room temperature to serve as a contact pad for electrical connection as shown in Fig. 6c.

In the fabrication, 400 *μ*m deep backside release trenches were achieved by silicon deep reactive ion etching (DRIE; SPTS Technologies Ltd., Ringland Way, Newport, UK). Before silicon etching, SiO_2_ on the wafer backside was cleaned by wet etching in BOE 6:1 at room temperature, followed by a deionized water rinse. Afterward, an Al_2_O_3_ hard mask was deposited by atomic layer deposition at 150 °C and patterned using an ICP-RIE system with 30 sccm BCl_3_ and 10 sccm of Cl_2_ gas at 1000 W ICP and 75 W RIE power. The DRIE was performed immediately after the mask patterning using the Bosch process at 3°C. The BOX layer was used as the stopping layer and the trench depth was confirmed by optical profilometry (NexviewTM NX2, Zygo Corp., CT, USA). Then, the sample was diced into an individual array with 32 active elements. The arrays were glued to a printed circuited board (PCB) and electrically connected via Au wire bonding. The PMUT was waterproofed using a 6 *μ*m thick parylene coating.

### B. PMUT CHARACTERIZATION

A custom printed circuit board (PCB) was designed with an on board dual-row 36 position header connector. The ground plane of the thin film array was connected to the ground pads of the PCB with conductive silver paint. Each of the 32 elements was wire-bonded to the excitation pad.

**FIGURE 7.**
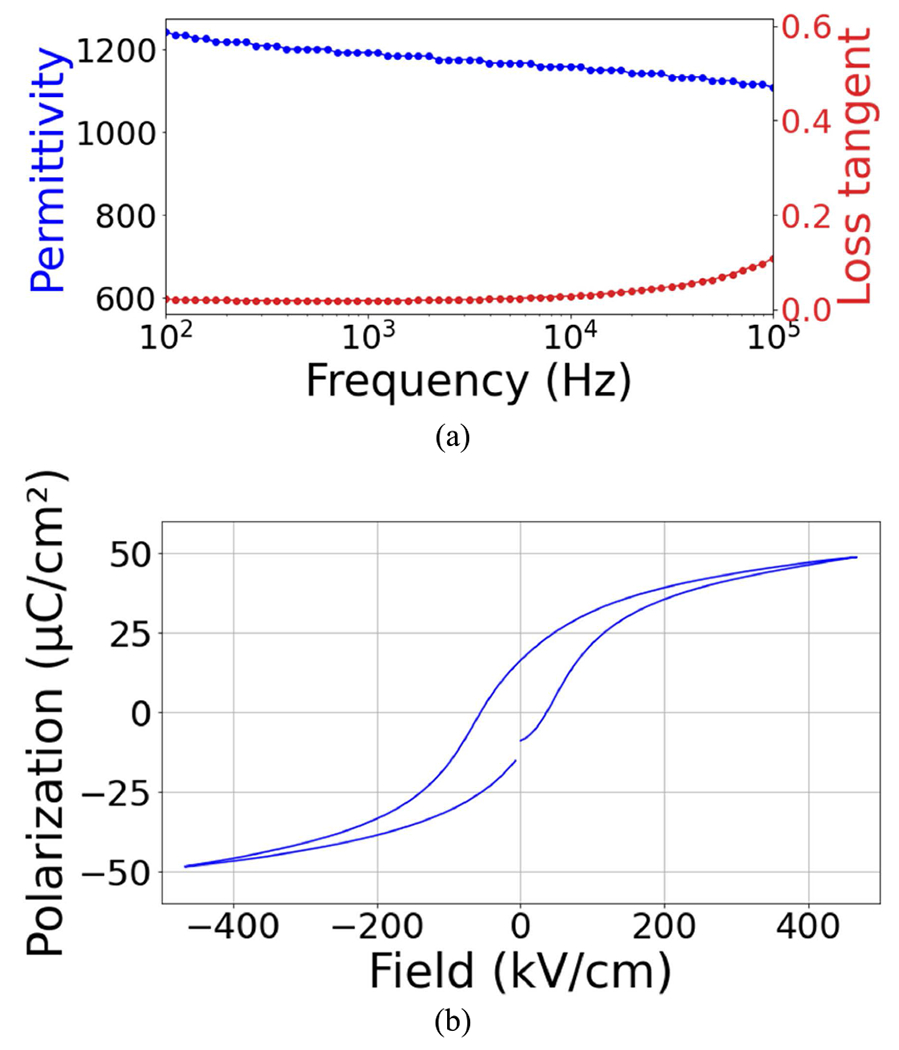
(a) The dielectric permittivity as a function of frequency showed a relative permittivity of 1210 ± 12 and dielectric loss of 1.9% ± 0.09% and (b) polarization-electric field hysteresis showed a remanent polarization, *P_r_*, and coercive fields, *E_c_*, of 16.2 *μ*C/cm^2^ and −54.9 and 39.2 kV/cm.

As shown in Fig. 7a, the relative permittivity and dielectric loss as a function of frequency from 100 Hz to 1 MHz with a 30 mV_AC_ excitation was measured using a Hewlett Packard 4284A LCR meter. The relative dielectric permittivity and loss tangent of 32 elements array was 1210 ± 12 and 1.9% ± 0.09%. A Radiant Multiferroic test analyzer was employed for analyzing the polarization-electric field hysteresis loop (P–E), which is shown in Fig. 7b. The P–E loop shows the remanent polarization (*P_r_*) of 16.2 *μ*C/cm^2^ and coercive fields (*E_c_*) of −54.9 and 39.2 kV/cm.

For driving the array, a 32 channel beamformer circuit (TX7332EVM, Texas Instruments, Dallas, TX, USA) was used as described in [30]. In short, the beamformer provides a maximum of 6 W power, 200 V_PP_ pulses, and a delay range of 0-40 *μ*s with 5 ns resolution.

**FIGURE 8.**
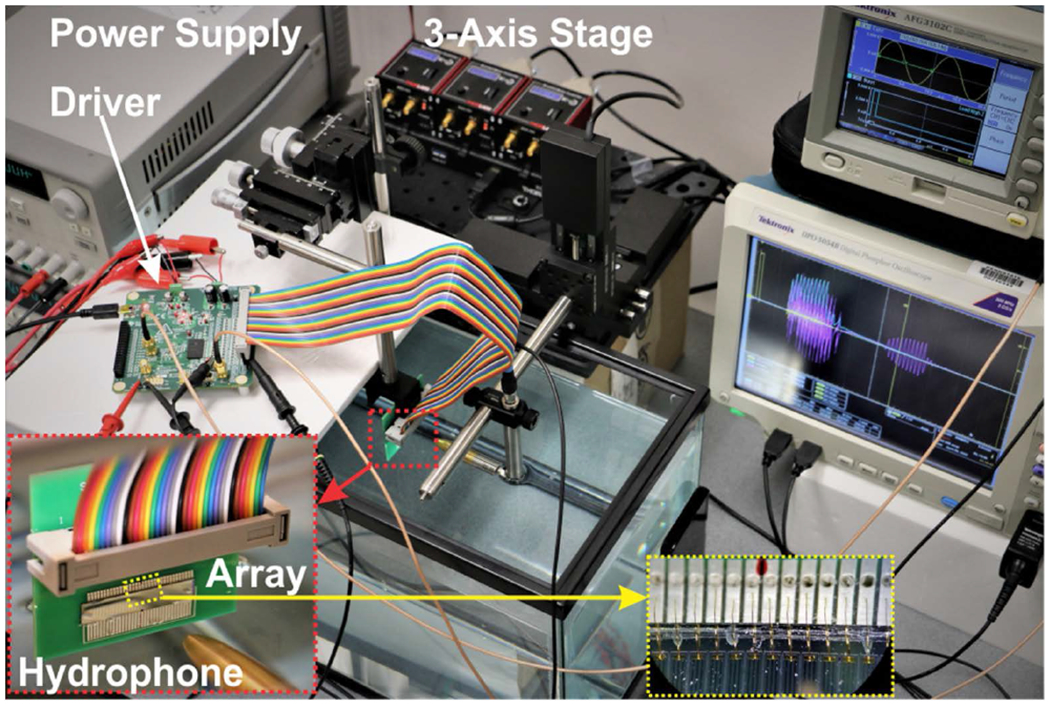
Beam profile measurement setup. Array assembly on a custom PCB with dual-row 36 position header connected to the driver board with a 36 position flat cable. Array elements were wire-bonded to the excitation pads.

Fig. 8 shows the beam measurement setup, including the beamformer circuit (with an interface), 3-axis motorized translation stage (MTS50/M-Z8, Thorlabs, Newton, NJ), calibrated hydrophone (pressure sensitivity of 48.2 nV/Pa at 1.4 MHz) with preamplifier, and digital oscilloscope (for data digitization and acquisition). A custom MATLAB code was used to coordinate all the equipment and automate the US beam scanning process.

## IV. THIN FILM PHASED ARRAY MEASUREMENT RESULTS

**FIGURE 9.**
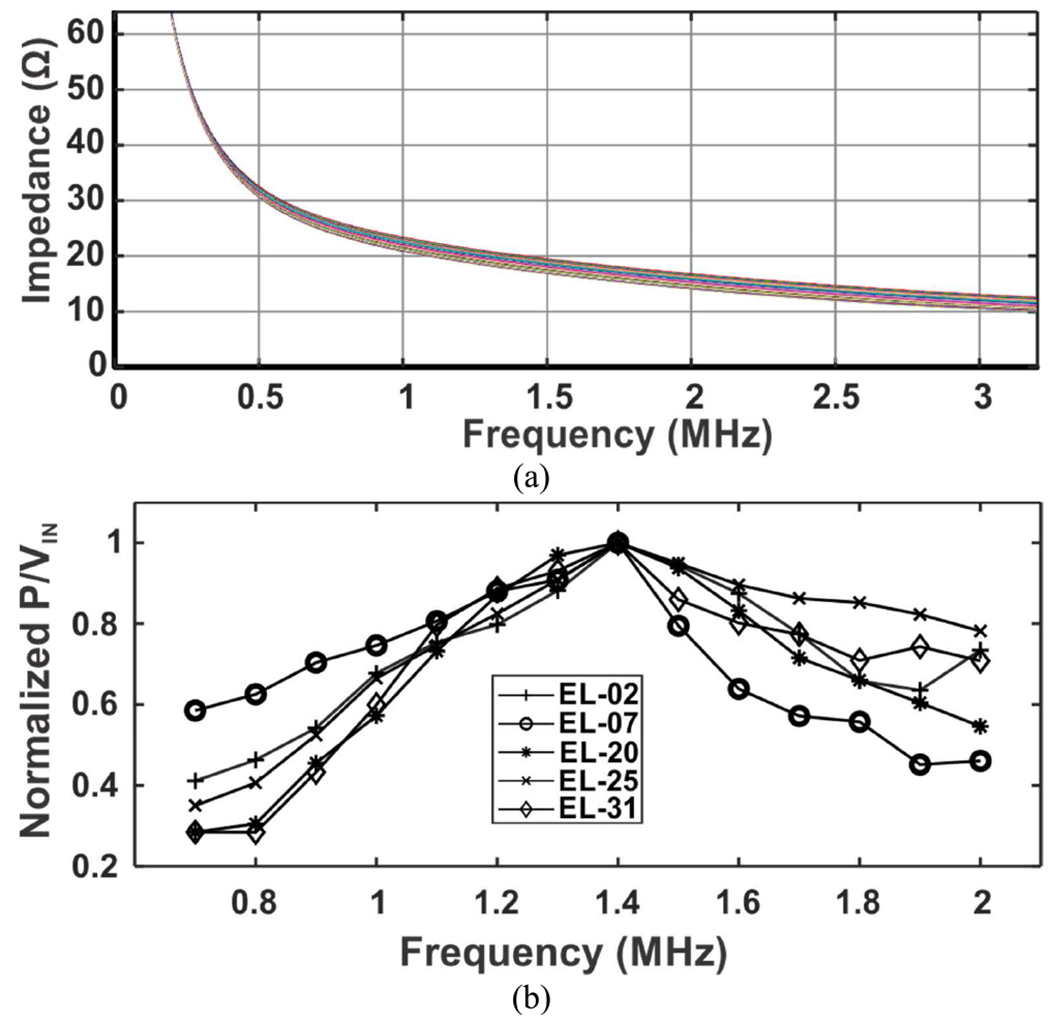
(a) Measured impedance of multiple elements. (b) Normalized output pressure over input voltage vs. frequency showing 1.4 MHz as the optimum driving frequency for multiple elements.

The electrical impedance of the individual elements was first measured at various frequencies ranging from 0.2 to 3.2 MHz, as shown in Fig. 9a. To find the optimum driving frequency, the ratio of the output pressure and the input voltage was measured as a function of frequency. Fig. 9b shows the normalized output pressure over input voltage vs. frequency for 5 different elements; 1.4 MHz was the optimum driving frequency. The deviation of resonant frequency relative to the calculated value may result from the differences in dimension between the calculation and fabricated device due to errors in micromachining such as the lithography misalignment and/or over etching. Thus, 1.4 MHz was selected as the driving frequency for all subsequent measurements.

**FIGURE 10.**
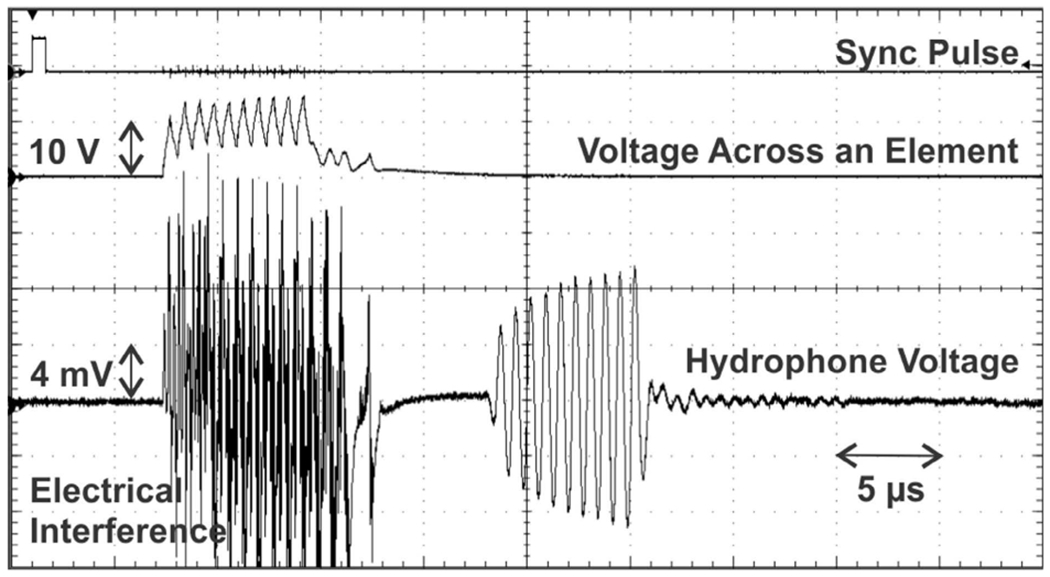
A hydrophone voltage waveform received from a beam formed at *F* = 20 mm (*θ_s_* = 0°) representing 45.9 kPa/V pressure output.

Fig. 10 shows a voltage waveform received by the hydrophone which represents a spatial peak pressure output of ~0.367 MPa (45.9 kPa/V). The corresponding beam (*F* = 20 mm and *θ*_*s*_ = 0°) was generated by the phased array being driven with 10 cycles of unipolar 1.4 MHz square pulses (8 V peak to peak). A smaller number of cycles was intentionally used for decoupling electrical interference.

**FIGURE 11.**
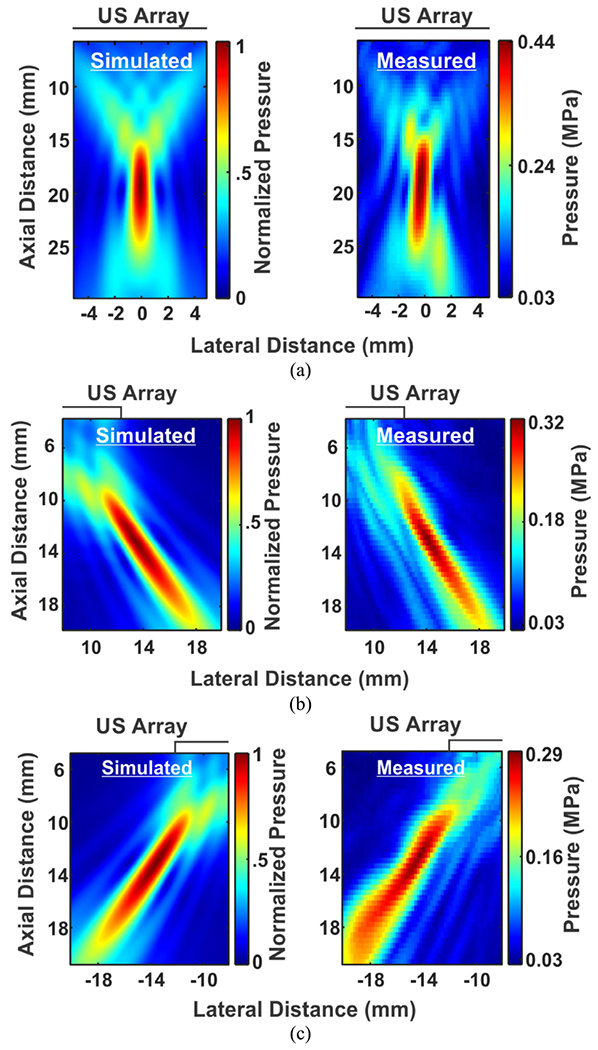
The 2D beam profiles of the phased array from simulations (left) and measurements (right) focused and steered at *F* = 20 mm and (a) *θ_s_* = 0°, (b) *θ_s_* = 45°, and (c) *θ_s_* = −45°.

Fig. 11a, 11b, and 11c present the 2D beam profiles from the simulation and measurement results at *F* = 20 mm with *θ*_*s*_ = 0°, 45°, and −45°, respectively. The simulated and measured axial resolutions at *θ*_*s*_ = 0°, 45°, −45° were 7.9, 9.9, 9.9 mm and 9.2, 10.3, 11.1 mm, respectively. For the lateral (*y*) resolution, the simulated results were 1.2, 1.3, 1.3 mm while the measurement results were 1, 1.3, 1.4 mm at *θ*_*s*_ = 0°, 45°, and −45°, respectively. The measured beam profiles closely matched the simulated ones. With 14.6 V driving voltage, the spatial peak pressure measured at *θ*_*s*_ = 0°, 45°, and −45° were 0.44 MPa, 0.33 MPa, and 0.29 MPa, respectively. Additionally, as expected, there was no significant off target high pressure spot.

**FIGURE 12.**
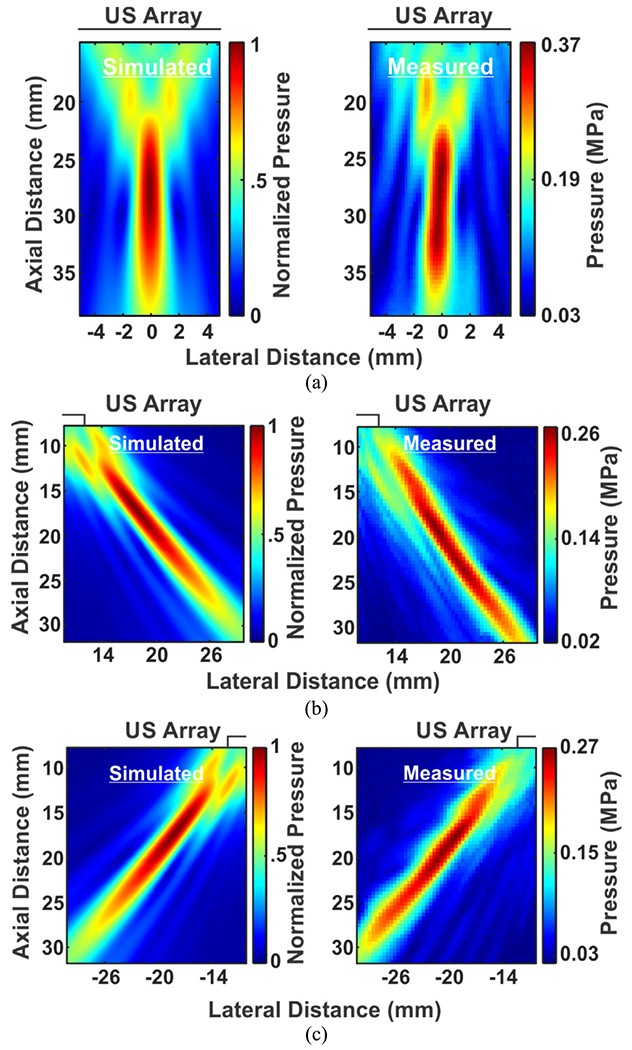
The 2D beam profiles of the phased array from simulations (left) and measurements (right) focused and steered at *F* = 30 mm and (a) *θ_s_* = 0°, (b) *θ_s_* = 45°, and (c) *θ_s_* = −45°.

Similarly, Fig. 12a, 12b, and 12c show the 2D beam profiles comparing the simulated and measured results with the beam focused at *F* = 30 mm. The simulated and measured axial (lateral) resolutions at *θ*_*s*_ = 0°, 45°, −45° were 14.1, 17.4, 17.4 mm (1.3, 1.9, 1.9 mm) and 13.6, 24.3, 22 mm (1.6, 2.2, 2 mm), respectively. As the targeted focal distance increased, the maximum acoustic pressure dropped to 0.37 MPa, 0.27 MPa, and 0.27 MPa at *θ*_*s*_ = 0°, 45°, and −45°, respectively.

**FIGURE 13.**
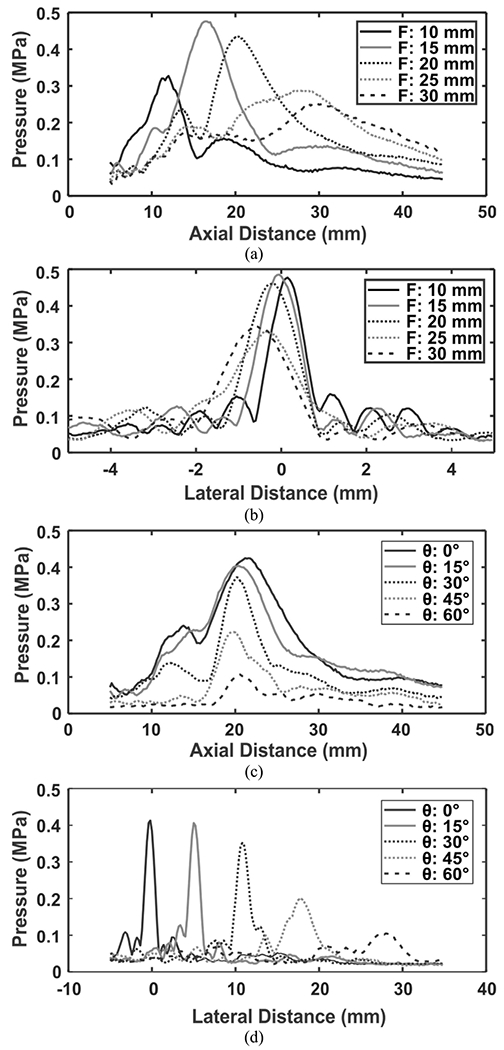
Measured 1D beam profiles focused and steered at different *F* and *θ_s_*. (a) Axial pressure profiles at different *F*, (b) lateral (*y*) pressure profiles at different *F*, (c) pressure profiles parallel to the *x* axis at different *θ_s_*, and (d) lateral (*y*) pressure profiles at different *θ_s_*.

Fig. 13a shows measured axial pressure profiles (***y*** = ***z*** = 0) of the beams focused at different focal distances (*F*). Fig. 13b shows measured lateral (***y***) pressure profiles of the beams at the axial distances where the peaks shown are corresponding to the maximum pressure output at different *F* from Fig. 13a. It is obvious that at *F* = 30 mm, which exceeds the *F*_*max*_ of 20 mm, the beam becomes comparatively wider (indicative of poor lateral resolution). One can note that in this case a higher *N* (or larger array aperture, *D*) could be used to compensate for the poor resolution. Fig. 13c shows the measured pressure profiles parallel to the ***x*** axis (as defined in Fig. 1) of the beams focused at *F* = 20 mm and steered at 0° to 60°. Fig. 13d shows the measured lateral (***y***) pressure profiles of the beams focused at *F* = 20 mm and steered at 0° to 60°. Although the fabricated array was optimally driven at 1.4 MHz, whereas it was designed for 1 MHz, the phase performance is still good showing no unwanted grating lobes for the maximum steering angle of 60°.

Table 2 summarizes the simulated and measured 2D beam characteristics including the axial and lateral resolution, maximum peak-to-peak pressure, and the corresponding *I_SPPA_* for the beams focused at *F* = 20 and 30 mm and steered at *θ*_*s*_ = 0°, 45°, −45°. *I_SPPA_* was calculated using the pulse intensity integral of the spatial peak pressure waveform as described in [9]. It should be noted that these results were obtained without the effect of a skull. The effect of a rat’s skull on the US beam shape and pressure of a bulk array has already been discussed with measurement results in previous work [30]. Given that the output pressures from the PMUT demonstrated in this work are high, it is anticipated that the PMUT array can also provide useful neurostimulation through a rat skull.

## V. CONCLUSIONS

The design methodology of a US transducer array for neuromodulation application was applied to PMUT transducers, with a design goal of exceeding hundreds of kPa acoustic pressure within the operated voltage below 20 V. The linear 32-element phased array PMUT thin film with a 1.4 MHz bending-mode resonant frequency was designed and fabricated with 1.5 *μ*m thick {100} oriented PZT as the piezoelectric layer and 2 *μ*m of silicon as the passive elastic layer. With a commercial driver board, the 32-element array was driven using calculated time delays for beam focusing and steering at *F* = 20-30 mm and *θ*_*s*_ = 0°-60°. The phased array demonstrated a maximum peak-to-peak acoustic pressure output of 0.44 MPa, corresponding to an acoustic intensity (*I_SPPA_*) of 1.29 W/cm^2^, achieved at 20 mm focal depth with 14.6 V unipolar square pulses in an immersion test. This work presents a new route to achieve low voltage US transducers for neuromodulation. Such PMUT US phased arrays can be integrated with a CMOS integrated circuit to drive the transducers.

## Figures and Tables

**TABLE 1.** Optimized design parameters.

Parameters	Optimized US Array	Fabricated US Array
Sonication Frequency, *f*(MHz)	1	1.4
Target Focal Distance, *F* (mm)	20	20
Number of US Elements, *N*	32	32
US Array Aperture, *D* (mm)	24.9	~25.3
US Element Length, *L* (mm)	8.3	~8.3
US Element Width, *a* (μm)	680	~521
US Interelement Spacing, *d* (mm)	0.78	~0.795
Steering Angle, *θ_s_* (deg)	±60	±60
Kerf, *kerf* (μm)	96.5	~272

**TABLE 2.** Summary of simulated and measured 2D beam profiles of the thin film array.

Sim / Meas	Medium	Focal Dis., *F* (mm)	Steering Angle, *θ_s_* (deg)	Axial Res. (mm)	Lateral (*y*) Res. (mm)	*Peak-to-Peak US Pressure (MPa)	US Intensity, *I_SPPA_* (W/cm^2^)
Sim	Brain Tissue	20	0	7.9	1.2	1	-
Meas	Water			9.2	1	0.44	1.29
Sim	Brain Tissue		45	9.9	1.3	0.74	-
Meas	Water			10.3	1.3	0.33	0.76
Sim	Brain Tissue		−45	9.9	1.3	0.74	-
Meas	Water			11.1	1.4	0.29	0.55
Sim	Brain Tissue	30	0	14.1	1.3	0.75	-
Meas	Water			13.6	1.6	0.37	0.91
Sim	Brain Tissue		45	17.4	1.9	0.54	-
Meas	Water			24.3	2.2	0.27	0.45
Sim	Brain Tissue		−45	17.4	1.9	0.54	-
Meas	Water			22	2	0.27	0.46

* Simulated peak-to-peak US pressure values are normalized to that of *F* = 20 mm and *θ_s_* = 0°.
